# Mitogenome types of two *Lentinula edodes* sensu lato populations in China

**DOI:** 10.1038/s41598-019-45922-5

**Published:** 2019-07-01

**Authors:** Xiaoxia Song, Yan Zhao, Chunyan Song, Mingjie Chen, Jianchun Huang, Dapeng Bao, Qi Tan, Ruiheng Yang

**Affiliations:** Institute of Edible Fungi, Shanghai Academy of Agricultural Sciences, Shanghai, 201403 People’s Republic of China

**Keywords:** Fungal genomics, Applied microbiology

## Abstract

China has two populations of *Lentinula edodes* sensu lato as follows: *L. edodes* sensu stricto and an unexcavated morphological species respectively designated as A and B. In a previous study, we found that the nuclear types of the two populations are distinct and that both have two branches (A1, A2, B1 and B2) based on the internal transcribed spacer 2 (ITS2) sequence. In this paper, their mitogenome types were studied by resequencing 20 of the strains. The results show that the mitogenome type (mt) of ITS2-A1 was mt-A1, that of ITS2-A2 was mt-A2, and those of ITS2-B1 and ITS2-B2 were mt-B. The strains with heterozygous ITS2 types had one mitogenome type, and some strains possessed a recombinant mitogenome. This indicated that there may be frequent genetic exchanges between the two populations and both nuclear and mitochondrial markers were necessary to identify the strains of *L. edodes* sensu lato. In addition, by screening SNP diversity and comparing four complete mitogenomes among mt-A1, mt-A2 and mt-B, the *cob*, *cox3*, *nad2*, *nad3*, *nad4*, *nad5*, *rps3* and *rrnS* genes could be used to identify mt-A and mt-B and that the *cox1, nad1* and *rrnL* genes could be used to identify mt-A1, mt-A2 and mt-B.

## Introduction

*Lentinula edodes* (Berk.) Pegler belongs to *Lentinula*, Omphalotaceae, Agaricales, Agaricomycetes, Basidiomycota, Fungi^1^. Its morphological characteristics were first described by Berkeley in 1877 based on a very poor specimen purchased from a shop in Japan^2^ and more than 12 names have been used to represent the species^3,4^. In the latter half of the 20^th^ century, the species was mainly placed into the genus *Lentinus*, as was proposed by Singer^5^. Until 1975, Pegler found that *L. edodes* had a monomictic hyphal system with generative hyphae, whereas *Lentinus* had a dimitic system with generative hyphae and skeleton-ligative hyphae. Therefore, this species was placed into the genus *Lentinula*. This classification is supported by many molecular phylogenetic studies^6,7^.

According to the morphological characteristics and geographical distribution, two species of *Lentinula* (*L. lateritia* and *L. novaezelandiae*) were distinct from *L. edodes*. However, mating compatibility studies have demonstrated that these three-morphological species are interfertile^8^. Many molecular phylogenetic studies confirm that these morphological species should belong to a biological species^9–15^. Some mycologists regard this biological species as Asian-Australasian populations of *Lentinula* according to their geographical distribution, and this species is also known as shiitake, an informal term in Japan^12,16^. However, according to nomenclatural priority, the biological species should be named *L. edodes*. To distinguish between the two *L. edodes* species, the biological species has been named *L. edodes* sensu lato^17^ and the morphological species has been named *L. edodes* sensu stricto^12^.

The nuclear ribosomal internal transcribed spacer (ITS) region has been used as a universal DNA barcode marker for fungi^18^. Hibbett *et al*.^12,13^ and Xu *et al*.^16^ used the ITS region to study the phylogenetic relationship of *L. edodes* sensu lato and found that there should be four distinct lineages of *L. edodes* sensu lato. In addition to *L. edodes* sensu stricto (in North-east Asia), *L. lateritia* (in South-east Asia and Australasia) and *L. novaezelandiae* (in New Zealand), there should be another unexcavated morphological species (in south-western East Asia). In addition, *L. edodes* sensu stricto and *L. lateritia* both tend to dissimilate into two subgroups. China is the first country to begin cultivating *L. edodes* sensu lato^19^ and is an important genetic diversity center of *L. edodes* sensu lato^16^; the informal Chinese term for *L. edodes* sensu lato is Xianggu. According to the geographical distribution of the four distinct lineages of *L. edodes* sensu lato, China has two populations: *L. edodes* sensu stricto and an unexcavated morphological species.

The genomic ITS region contains three sequences: internal transcribed spacer 1 (ITS1), 5.8 S ribosomal DNA (5.8 S) and internal transcribed spacer 2 (ITS2), and exhibits an incomplete concerted evolution^20^. Compared with 5.8 S sequence, ITS1 and ITS2 sequences have comparable discriminating power and barcode gaps, and could be used to instead of the whole ITS region for discriminating many sister species^21,22^. In addition, because the relatively short length of ITS2 (as compared to the whole ITS regions) allows Illumina and other next-generation sequencing technologies to obtain the whole ITS2 sequence in paired-end sequencing, using ITS2 sequence alone does have an advantage^22^. In the previous work, we found that ITS1 and ITS2 sequences were similar for discriminating two populations of *L. edodes* sensu lato in China, and that ITS2 could be also used for discriminating the branches of two populations^23^. Based on their ITS2 sequences, *L. edodes* sensu stricto (marked as ITS2-A) contained two branches (ITS2-A1 and ITS2-A2), and the unexcavated morphological species (marked as ITS2-B) also contained two branches (ITS2-B1 and ITS2-B2). In addition, we found that many strains were heterozygous ITS2 types, such as A1 + A2, A2 + B1, A2 + B2 and B1 + B2, which came from their heterokaryons.

The strain of *L. edodes* sensu lato is a filamentous fungi, and its hyphal cell harbors two nuclei that are inherited from both parents and one cytoplasm that is inherited from mother^24,25^. The mitochondrion is the powerhouse of the cytoplasm and contains its own genetic material, and the mitochondrial genome (mitogenome) has been widely accepted as an effective evolutionary marker in fungi^26–29^. Because the ITS2 sequence is located in the nuclear genome, one question arises as to whether the two populations of *L. edodes* sensu lato with different ITS2 types belong to identical or different mitogenome types (mt). Therefore, we selected 20 strains with different ITS2 types to answer this question. The specific research contents are as follows: 1) Sequence the complete mitogenome of L135 as the reference genome. 2) Study the mitogenome types of the two *L. edodes* sensu lato populations in China by resequencing the mitogenomes of 19 strains with different ITS2 types. 3) Study the SNP and indel diversity among the different mitogenome types. 4) Screen suitable markers for identifying different mitogenome types by sequencing the complete mitogenome of one strain that belonged to different mitogenome type from that of L135, and these two complete mitogenomes were aligned with the other two complete mitogenomes of *L. edodes* sensu lato (NC_018365.1 and KY217797.1) from the NCBI Organelle Genome Database.

## Results

### The reference mitogenome of L135

**Figure 1:**
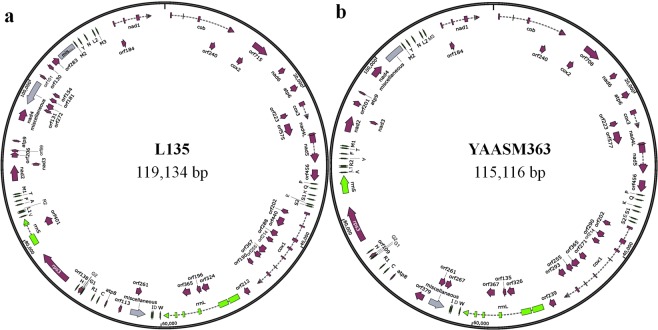
The mitogenome maps of L135 and YAASM363. (**a**) The mitogenome map of L135. (**b**) The mitogenome map of YAASM363. The protein-coding genes (wine red), rRNA genes (green), tRNA genes (bottle green), miscellaneous features (gray), introns (dotted line), hypothetical protein-coding genes (orf + number), and strand coding direction (arrow direction: clockwise-sense, anticlockwise-antisense) are shown.

The mitogenome of L135 is a circular DNA molecule that is 119,134 bp in size with a GC content of 30.77%. It consists of 15 conserved protein-coding genes, 2 rRNA genes, 26 tRNA genes, 30 hypothetical protein-coding genes (10 in the introns of 4 conserved protein-coding genes, 4 in the introns of 2 rRNA gene and 16 in intergenic regions) and 3 miscellaneous features (Fig. 1a).

The 15 conserved protein-coding genes include genes that encode NADH dehydrogenase subunits (*nad1*, *nad2*, *nad3*, *nad4*, *nad4L*, *nad5* and *nad6*), apocytochrome b (*cob*), cytochrome c oxidase subunits (*cox1*, *cox2* and *cox3*), ATP synthase A chain subunits (*atp6*, *atp8* and *atp9*) and ribosomal protein S3 (*rps3*) (Table 1). Except for *atp8*, all other 14 genes were encoded on the sense strand (Fig. 1a). All 15 conserved protein-coding genes had the start codon ATG. However, 11 of the 15 genes had TAA as the stop codon, while the remaining four (*cox2*, *cox3*, *nad4* and *nad5*) had TAG as the stop codon. In addition, 5 conserved protein-coding genes harbored 15 introns as follows: *cob* (3 introns), *cox3* (1 intron), *nad5* (1 intron), *cox1* (7 introns), and *nad1* (3 introns); in addition, 10 introns harbored the 10 following hypothetical protein-coding genes: 1 intron of *cob*, 1 intron of *cox3*, 7 introns of *cox*1 and 1 intron of *nad1*. The 10 intronic hypothetical protein-coding genes were encoded on the sense strand (Fig. 1a).The large-subunit rRNA (*rrnL*) gene had 5 introns and 2 introns contained 3 intronic hypothetical protein-coding genes. The small-subunit rRNA (*rrnS*) gene had 1 intron that contained 1 intronnic hypothetical protein-coding gene. The 2 rRNA genes were encoded on the sense strand. The 4 intronic hypothetical protein-coding genes of 2 rRNA were encoded on the sense strand (Fig. 1a).

**Table 1:** Protein-coding gene information of the L135 mitogenome.

Gene	Position	Product
*cob*	712–867, 2340–2366, 4254–4481, 8351–9109	Apocytochrome b
orf240	6886–7608	Intronic hypothetical protein of *cob*
*cox2*	11477–12235	Cytochrome c oxiddase subunit 2
orf715	12024–14171	Intergenic hypothetical protein
*nad6*	16635–17261	NADH dehydrogenase subunit 6
*atp6*	19254–20021	ATP synthase A chain subunit 6
*cox3*	21577–22041, 23491–23856	Cytochrome c oxidase subunit 3
orf223	22081–22752	Intronic hypothetical protein of *cox3*
orf575	23660–25387	Intergenic hypothetical protein
*nad4L*	25711–25977	NADH dehydrogenase subunit 4L
*nad5*	25980–26915, 28324–29436	NADH dehydrogenase subunit 5
orf456	30354–31724	Intergenic hypothetical protein
*cox1*	35868–36101, 37627–37776, 39508–39837, 40811–41014, 42270–42401, 43865–43915, 45013–45210, 46653–46955	Cytochrome c oxidase subunit 1
orf202	36456–37064	Intronic hypothetical protein of *cox1*
orf440	37780–39102	Intronic hypothetical protein of *cox1*
orf288	39907–40773	Intronic hypothetical protein of *cox1*
orf214	41165–41809	Intronic hypothetical protein of *cox1*
orf367	42731–43834	Intronic hypothetical protein of *cox1*
orf255	43934–44701	Intronic hypothetical protein of *cox1*
orf190	45514–46086	Intronic hypothetical protein of *cox1*
orf212	48304–48942	Intergenic hypothetical protein
orf324	53014–53988	Intronic hypothetical protein of *rrnL*
orf196	54200–54790	Intronic hypothetical protein of *rrnL*
orf365	55819–56916	Intronic hypothetical protein of *rrnL*
orf261	62988–63773	Intergenic hypothetical protein
orf113	65115–65456	Intergenic hypothetical protein
*atp8*	67101–67259	ATP synthase A chain subunit 8
orf138	70657–71073	Intergenic hypothetical protein
*rps3*	72924–77330	Ribosomal protein S3
orf401	80332–81537	Intronic hypothetical protein *rrnS*
*nad2*	87398–89332	NADH dehydrogenase subunit 2
*nad3*	89332–89715	NADH dehydrogenase subunit 3
orf206	90359–90979	Intergenic hypothetical protein
orf99	91060–91359	Intergenic hypothetical protein
*atp9*	92336–92557	ATP synthase A chain subunit 9
*nad4*	95114–96577	NADH dehydrogenase subunit 4
orf131	97316–97711	Intergenic hypothetical protein
orf272	97818–98636	Intergenic hypothetical protein
orf181	98998–99543	Intergenic hypothetical protein
orf154	99933–100397	Intergenic hypothetical protein
orf101	101124–101429	Intergenic hypothetical protein
orf130	102620–103012	Intergenic hypothetical protein
orf283	103451–104302	Intergenic hypothetical protein
*nad1*	112551–112703,113911–114054, 115558–115917, 117078–117437	NADH dehydrogenase subunit 1
orf184	112770–113324	Intronic hypothetical protein of *nad1*

The 26 tRNA genes coded for 20 common amino acids and were encoded on the sense strand (Fig. 1a). Of these 21 tRNA genes varied in size from 71 bp to 76 bp and could be folded into the typical cloverleaf structure. However, 5 other tRNA genes, including the tRNAs for the amino acids S (34080-34187), S (34964–35047), L (83281–83369), Y (107743–107826) and L (109774–109856), varied in size from 83 bp to 89 bp and had an additional variable loop (see Supplementary File 1).

The remaining regions without annotation were intergenic regions and 16 intergenic hypothetical protein-coding genes were located in these regions. Except for 6 intergenic hypothetical protein-coding genes (orf261, orf131, orf272, orf181, orf154 and orf130), the other 10 intergenic hypothetical protein-coding genes were encoded on the sense strand. In addition, the 3 miscellaneous features may encode mitochondrial plasmid DNA polymerase and RNA polymerase (Fig. 1a).

### Mitogenome types of the 20 strains with different ITS2 types

**Figure 2:**
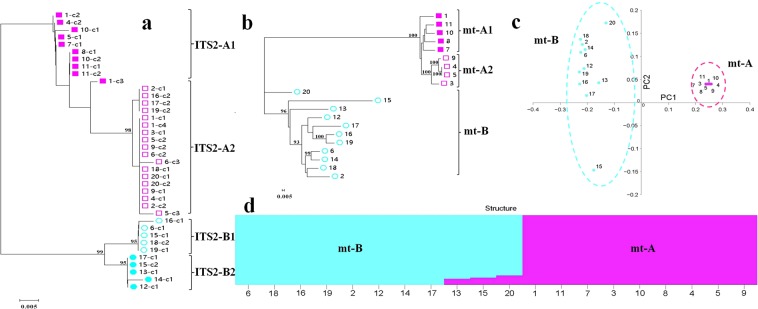
Population analysis of ITS2 and the mitogenome of 20 strains. (**a**) A NJ tree was constructed using the ITS2 data. Different ITS2 sequences of each strain were marked as c1, c2, c3 and c4 (in order). (**b**) A NJ tree was constructed the using the SNP data. (**b**) PCA. (**c**) Population structure. The number of strains was the same as those listed in Table 2. Population A: purple; Population B: blue.

According to the results of Song *et al*.^23^, the 20 strains had 38 ITS sequences (Table 2). Therefore, 38 ITS2 sequences were extracted from the 38 ITS sequences to construct their neighbor-joining (NJ) tree. The 38 ITS2 sequences were divided into two lineages (bootstrap support = 99%): ITS2-A and ITS2-B. Each lineage was subdivided into 2 branches (bootstrap support ≥95%): ITS2-A1 and ITS2-A2, and ITS2-B1 and ITS2-B2 (Fig. 2a).

**Table 2:** Summary of 20 strains.

Number	Strain	Source (Province)	Wild or cultivar	GenBank of ITS sequence*
1	Cr01	Fujian	cultivar	KY494433-KY494436
2	EFISAAS0376	Yunnan	wild	KY494551,KY494552
3	EFISAAS5052	Guizhou	wild	KY494553
4	EFISAAS5053	Guizhou	wild	KY494554,KY494555
5	EFISAAS5054	Guizhou	wild	KY494556-KY494558
6	EFISAAS5145	Yunnan	wild	KY494561-KY494563
7	EFISAAS5143	Jilin	wild	KY494559
8	EFISAAS5146	Liaoning	wild	KY494564
9	Guangxiang No.9	Guangdong	cultivar	KY494445-KY494446
10	L135	Fujian	cultivar	KY494470,KY494471
11	L808	Zhejiang	cultivar	KY494478,KY494479
12	YAASM296	Yunnan	wild	KY494583
13	YAASM298	Yunnan	wild	KY494584
14	YAASM300	Yunnan	wild	KY494588
15	YAASM301	Yunnan	wild	KY494589,KY494590
16	YAASM359	Yunnan	wild	KY494596,KY494597
17	YAASM363	Yunnan	wild	KY494598,KY494599
18	YAASM1515	Yunnan	wild	KY494611,KY494612
19	YAASM2321	Sichuan	wild	KY494634,KY494635
20	YAASM2323	Sichuan	wild	KY494636,KY494637

Notes: *cites from Song *et al*.^23^.

Based on the mitogenome of L135, the remaining 19 strains (Table 2) were resequenced, and their mitogenomes were assembled. The raw clean reads of the 19 strains varied from 6.69 million to 41.26 million with Q30 > 91%, and the average depths of the 19 strains were more than 544-fold (see Supplementary Table S1). Compared to the mitogenome coverage of L135, the mitogenome coverage of the 19 strains averaged approximately 95%. A total of 12,321 SNPs and 2,272 indels were identified (Table 3).

**Table 3:** SNPs and indels among the mitogenomes of the 19 resequenced strains aligned with L135.

Population type	Strain	CDS		Intron		Intergenic region		Total	
		SNPs	InDels	SNPs	InDels	SNPs	InDels	SNPs	InDels
A1	Cr01	27	6	21	4	97	25	145	35
	EFISAAS5143	19	5	18	1	132	23	169	29
	EFISAAS5146	23	5	19	1	115	34	157	40
	L808	16	3	8	1	85	21	109	25
	*Average*	*21*	*4*	*16*	*1*	*107*	*25*	*145Bb*	*32Bc*
A2	EFISAAS5052	23	9	25	6	179	42	227	57
	EFISAAS5053	27	7	20	2	169	48	216	57
	EFISAAS5054	27	7	21	2	172	42	220	51
	Guangxiang No.9	26	8	22	3	180	42	228	53
	*Average*	*25*	*7*	*22*	*3*	*175*	*43*	*222Bb*	*54Bb*
B	EFISAAS0376	150	17	206	30	638	126	994	173
	EFISAAS5145	142	14	223	37	668	133	1033	184
	YAASM296	145	15	203	29	609	124	957	168
	YAASM298	134	15	219	34	546	114	899	163
	YAASM300	143	14	211	38	614	126	968	178
	YAASM301	139	15	216	33	663	123	1018	171
	YAASM359	143	14	197	29	678	140	1018	183
	YAASM363	136	12	211	31	644	131	991	174
	YAASM1515	152	15	219	40	662	133	1033	188
	YAASM2321	147	15	228	33	729	149	1104	197
	YAASM2323	151	16	202	34	482	96	835	146
	*Average*	*143*	*14*	*212*	*33*	*630*	*126*	*986Aa*	*175Aa*

Notes: The different capital letters indicate significant differences at the 0.01 level, and the different lowercase letters indicate significant differences at the 0.05 level.

An NJ tree revealed that the mitogenomes of 20 strains belonged to two distinct lineages (bootstrap support = 100%): mt-A and mt-B; mt-A was subdivided into two branches (bootstrap support = 100%): mt-A1 and mt-A2 (Fig. 2b). The principal component analysis (PCA) (Fig. 2c) and population structure analyses (Fig. 2d) confirmed the results of the NJ tree. However, the 3 strains (YAASM298, YAASM301 and YAASM2323) were recombinant mitogenome types between mt-A and mt-B. Because most of their SNPs belonged to mt-B, these 3 strains were included in mt-B.

### SNP and indel diversity among mt-A1, mt-A2 and mt-B

By testing the considerable differences in the SNPs and indels among mt-A1, mt-A2 and mt-B, the number of SNPs and indels in mt-B was considerably higher than that in mt-A1 and mt-A2. The number of indels in mt-A2 was considerably higher than that in mt-A1. By testing the SNP diversity in the CDS region among the mitogenomes of the 19 resequenced strains aligned with L135 (see Supplementary Table S2), the mitogenomes of 18 strains, except for EFISAAS5146, had more synonymous mutations than nonsynonymous mutations in the defined CDS regions of the 15 conserved protein-coding genes, and all 19 strains had more nonsynonymous mutations than synonymous mutations in the CDS regions of the 31 hypothetical protein-coding genes. The number of synonymous and nonsynonymous mutations in mt-B was different that those of mt-A1 and mt-A2.

**Figure 3:**
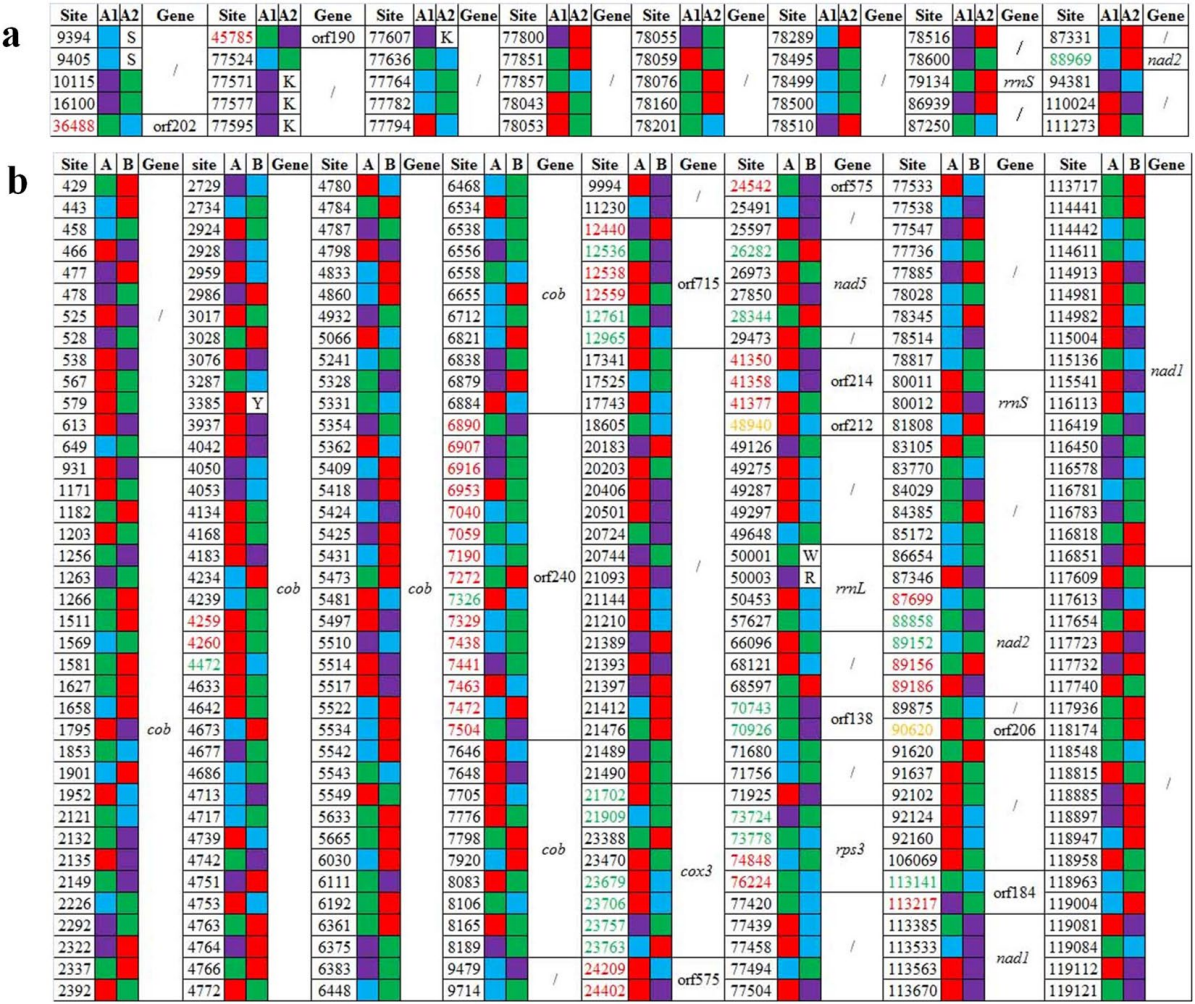
SNP differences among the different mitogenome types. (**a**) SNP differences between mt-A1 and mt-A2. (**b**) SNP differences between mt-A and mt-B. Site (red): nonsynonymous SNP in the CDS; Site (green): synonymous SNP in the CDS; Site (yellow): stoploss SNP in the CDS. DNA: adenine (green), thymine (red), guanosine (purple), cytosine (blue). Oligo DNA: K (guanosine/thymine), S (guanosine/cytosine), Y (thymine/cytosine), W (adenine/thymine) and R (adenine/guanosine)./indicates intergenic regions.

To screen some markers for identifying mt-A1, mt-A2 and mt-B, the different SNPs among mt-A1, mt-A2 and mt-B were tested. There were 40 different SNPs between mt-A1 and mt-A2 (Fig. 3a) with 36 SNPs in the intergenic region, 2 SNPs in the 2 intronic hypothetical protein-coding genes of *cox1* (orf202 and orf190), 1 SNP in *rrnS*, and 1 stoploss in the *nad2* CDS. There were 304 different SNPs between mt-A and mt-B (Fig. 3b) with 137 SNPs in *cob* [18 SNPs in its intronic hypothetical protein-coding gene (orf240) and 119 SNPs in the intron], 99 SNPs in the intergenic region and 13 SNPs in the intergenic hypothetical protein-coding genes (6 SNPs in the orf715, 3 SNPs in the orf575, 2 SNPs in the orf138, 1 stoploss in the orf212 and 1 stoploss in the orf206), 24 SNPs in the *nad1* gene [22 SNPs in the intron, 2 SNPs in its intronic hypothetical protein-coding gene (orf184)], 8 SNPs in the *cox3* gene (6 SNPs in the CDS and 2 SNPs in the intron), 5 SNPs in the *nad2* CDS, 4 SNPs in the *rrnL* gene, 4 SNPs in *nad5* (2 SNPs in the CDS and 2 SNPs in the intron), 4 SNPs in the *rps3* CDS, 3 SNPs in the intronic hypothetical protein-coding gene of *cox1* (orf214), 3 SNPs in the *rrnS* gene.

### Suitable markers for identifying mt-A1, mt-A2 and mt-B

**Figure 4:**
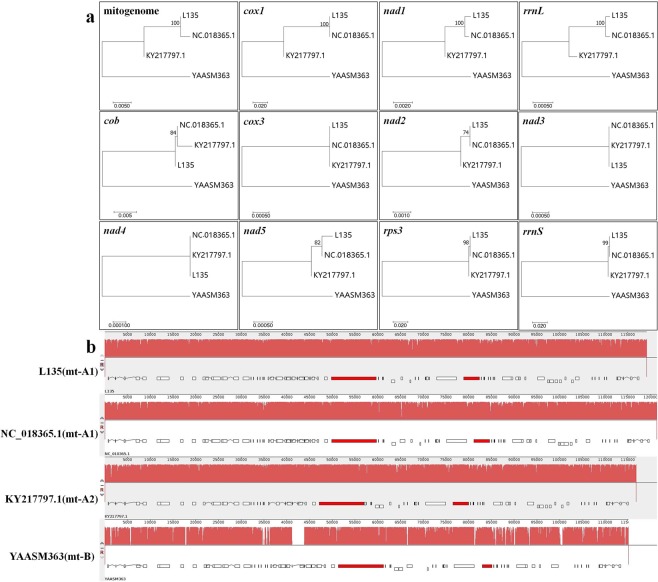
NJ trees and synteny analysis of the complete mitogenomes among L135, NC_018365.1, KY217797.1 and YAASM363. (**a**) NJ trees of the four complete mitogenomes based on different sequences. (**b**) Synteny analysis of the four complete mitogenomes.

To screen some markers for identifying mt-A1, mt-A2 and mt-B, the complete mitogenome of YAASM363 in mt-B was also tested. In addition, the two complete mitogenomes of *L. edodes* sensu lato (NC_018365.1 and KY217797.1) that were released in the NCBI database were also used in phylogenetic analysis (Fig. 4a mitogenome). The mitogenome of NC_018365.1 had a high homology with that of L135 (bootstrap support = 100%) and should belong to mt-A1. The KY217797.1 strain was collected by us in Guizhou Province and was identified as EFISAAS5052. Therefore, the mitogenome of KY217797.1 belonged to mt-A2.

The NC_018365.1 mitogenome had a good synteny with the L135 mitogenome in gene number and arrangement (Fig. 4b and Supplementary Table S3). The total length and GC content of the NC_018365.1 mitogenome were 121,394 bp and 30.70%, respectively. Based on the mitogenome annotation of NC_018365.1 from the NCBI database, some hypothetical protein-coding genes and *rrnL* introns of NC_018365.1 were determined by homology comparison with the mitogenome of L135 (see Supplementary Table S3). The mitogenome of NC_018365.1 consisted of 15 conserved protein-coding genes, 2 rRNA genes, 26 tRNA genes, 30 hypothetical protein-coding genes (10 in the introns of 4 conserved protein-coding genes, 4 in the introns of 2 rRNA gene and 16 in intergenic regions) and 3 miscellaneous features (see Supplementary Table S3).

The total length and GC content of the KY217797.1 mitogenome were 116,819 bp and 30.75%, respectively. Because the mitogenome annotation of KY217797.1 given in the NCBI database was poor, we reannotated it. It consists of 15 conserved protein-coding genes, 2 rRNA genes, 26 tRNA genes, 24 hypothetical protein-coding genes (4 in the introns of 4 conserved protein-coding genes, 4 in the introns of 2 rRNA gene and 16 in intergenic regions) and 3 miscellaneous features (see Supplementary Table S3). The biggest difference between KY217797.1 and L135 mitogenomes was the *cox1* gene, which missed two introns and 6 intronic hypothetical protein-coding genes in the KY217797.1 mitogenome (Fig. 4b and Supplementary Table S3).

The total length and GC content of the YAASM363 mitogenome were 115,116 bp and 31.76%, respectively (Fig. 1b). This mitogenome was found to contain 15 conserved protein-coding genes, 2 rRNA genes, 26 tRNA genes, 22 hypothetical protein-coding genes (10 in the introns of 4 conserved protein-coding genes, 3 in the introns of 1 rRNA gene and 9 in intergenic regions) and 2 miscellaneous features (Fig. 1b and Supplementary Table S3). The mitogenome of YAASM363 had many insertions, deletions and alternative splicing regions similar to those of L135 as follows: 1) *cox3*-exon1 of YAASM363 had an exon insertion. 2) *cox1*-exon2 and *cox1*-exon3 of L135 combined into *cox1*-exon2 in YAASM363, and *cox1*-exon5 of L135 broke up into *cox1*-exon4 and *cox1*-exon5 in YAASM363; therefore, the intronic hypothetical protein-coding gene (orf440) was lacking in YAASM363. 3) *rrnS* had no intron or the intronic hypothetical protein-coding gene (orf401) in YAASM363. 5) Miscellaneous features and 7 intergenic hypothetical protein-coding genes near *nad4* (orf131, orf272, orf181, orf154, orf101, orf130 and orf283) were missing in YAASM363. The other insertions and deletions in the intronic and intergenic regions were not detailed in the paper.

The 15 conserved protein-coding genes and the 2 rRNA genes of these four mitogenomes were extracted for alignment (Fig. 4a). The *atp6*, *atp8*, *atp9*, *cox2*, *nad4L* and *nad6* genes were identical in four mitogenomes. The *cob*, *cox3*, *nad2*, *nad3*, *nad4*, *nad5, rps3* and *rrnS* genes, which were distinct between mt-B and two branches of mt-A and only a small degree of difference between mt-A1 and mt-A2, could be used as markers for identifying mt-A and mt-B. The *cox1*, *nad1* and *rrnL* genes of four mitogenomes were all considerably different and could be used as markers for identifying mt-A1, mt-A2 and mt-B.

## Discussion

There are two main strategies for acquiring a complete mitogenome. The first strategy is to isolate the mitochondrial DNA separately from the whole genomic DNA of an organism and to sequence the mitogenome directly^30^; the second strategy is to sequence the whole genome and to assemble the mitogenome based on a sequencing library^31^. In this paper, the complete mitogenomes of L135 and YAASM363 were assembled based on Illumina (450 bp) and PacBio (8–10 kb) sequencing data. Genomic resequencing is widely used to analyze the genetic diversity and structure of populations. Xiao *et al*.^32^ analyzed the genetic diversity and population structure of wild and cultivated strains of Chinese *L. edodes* sensu lato by resequencing their nuclear genomes based on Illumina sequencing data. The mapped rates of the nuclear genome in the whole genome varied from 85.01% to 97.63%. In this paper, we analyzed the mitogenome types of two *L. edodes* sensu lato populations in China by resequencing their mitogenomes with Illumina sequencing. The mapped rates of the mitogenome in the whole genome varied from 2.25% to 11.27%. The total mapped rates of the nuclear genome and mitogenome were near 100%. Therefore, the resequencing analysis in this paper was feasible.

Since the beginning of the fungal mitogenome project^33^, 347 fungal mitogenomes have been released in the NCBI Organelle Genome Database, 220 of which have been curated^28^. These fungal mitogenomes vary extensively in size and range from 12 kb to over 235 kb^27^; this variation is mainly caused by intronic and plasmid-derived regions^34^. They generally have the same set of core genes as follows: 15 conserved protein-coding genes (*nad1*, *nad2*, *nad3*, *nad4*, *nad4L*, *nad5*, *nad6*, *cob*, *cox1*, *cox2*, *cox3*, *atp6*, *atp8*, *atp9* and *rps3*), 2 rRNA genes (*rrnS* and *rrnL*) and tRNA genes^25,34^. In the NCBI database, two complete mitogenomes of *L. edodes* sensu lato have been released: NC_018365.1 and KY217797.1. Because they have distinct annotations in the NCBI database, we did not select them as the reference mitogenome. In this paper, we reannotated them again by comparing them with those of L135 and YAASM363. Finally, these four mitogenomes of *L. edodes* sensu lato had 15 conserved protein-coding genes, 2 rRNA genes and 26 tRNA genes, and showed complete synteny with each other in the order of these genes (Fig. 4b and Supplementary Table S3).

Variations in mitochondrial genome sizes among different strains within the same species have also been reported in many fungi^35^. In this paper, the four complete mitogenomes of *L. edodes* sensu lato were very similar in size, ranging from about 115 to 121 kb. The two mt-A1 strains (L135 and NC_018365.1) had the largest mitogenome size, followed by one mt-A2 strain (KY217797.1), and then one mt-B strain (YAASM363) (Fig. 4b). Introns are the major contributors to the mitogenome size variations of these four complete mitogenomes. For example, the length of *cox1* exon was identical (1602 bp) in four complete mitogenomes. But the whole *cox1* genes of two mt-A1 strains (L135 and NC_018365.1) were 11088 bp in length and both had 7 introns; that of one mt-A2 strain (KY217797.1) was 8391 bp in length and missed 2 introns; that of one mt-B strain (YAASM363) was 12029 bp in length and had 7 introns with alternative splicing of 3 exons (see Supplementary Table S3).

The proportion of nonsynonymous mutations to synonymous mutations is commonly estimated in evolutionary biology for testing hypotheses related to selective pressure^36^. In the CDS regions of 15 conserved protein-coding genes, the proportion of nonsynonymous mutations to synonymous mutations between L135 and 18 strains, with the exception of EFISAAS5146, was less than 1. the evolution of the 15 conserved protein-coding genes between L135 and the 18 strains, except for EFISAAS5146, was due to purifying (negative) selection. The *rps3* gene of EFISAAS5146 had 4 nonsynonymous mutations and 1 synonymous mutation that was different from L135 and did not fit the rule. Tracing the *rps3* gene of the other 18 strains, the strains of group B had high levels of nonsynonymous mutations that were different from L135. This implied that the *rps3* gene had a higher rate of evolution than the other 14 conserved protein-coding genes. In the CDS region of 30 hypothetical protein-coding genes, the proportion of nonsynonymous mutations to synonymous mutations between L135 and 19 strains was greater than 1. This suggested that the evolution of the 31 hypothetical protein-coding genes in L135 and the 19 strains was based on diversifying (positive) selection. For example, the putative protein of orf214 was similar to a homing endonuclease containing a LAGRIDADG_2 domain that promotes the mobility of the intron^37^.

The nuclear and mitochondrial genetic relationships of the wild *L. edodes* sensu lato strains are highly associated with their geographic distribution^9,32^. ITS2-A1 is mainly distributed in northern Asia (Russia, Korea, Japan and northeastern and northwestern China) and ITS2-A2 is mainly distributed in southern Asia (pan-mid-southern China and Thailand). ITS2-B is mainly distributed in the southwest of eastern Asia (western China, India and Nepal)^12,13,16,23^. Tracing the source and parent information of the 20 strains (Table 2), there should be a correspondence between ITS2 and the mitogenome types in each population. The mitogenome type (mt) of each strain is as follows: ITS2-A1 was mt-A1, ITS2-A2 was mt-A2, and ITS2-B1 and ITS2-B2 were mt-B. However, 2 strains (EFISAAS0376 and YAASM2323) with the ITS2-A2 type that belonged to mt-B did not fit the rule. The best explanation is that these 2 strains may have exchanged their mitochondria with the other mitogenome type mt-B strains. This explanation needs to be validated. In addition, during the mon-mon crossing of two compatible homokaryons mitogenome recombination is easily found at the junction of two homokaryons^24,38,39^. The 3 strains with recombinant mitogenomes may be the offspring of mt-A and mt-B, and their SNPs from mt-A were analyzed (see Supplementary Fig. S1). These results indicate that there are frequent genetic exchanges between the two populations of *L. edodes* sensu lato in China.

Many studies have shown that the classification of the same species is considerably differentiated in both the mitochondrial and nuclear genes^40,41^. In animal, most species have shown a greater rate of evolution in mitochondrial genes, but the opposite is true for most plants and most fungi populations^35^. In the paper, we also calculated the percentage of plasmorphism site (pplymorphism rate) in ITS2 and the whole mitogenome between L135 and the other 19 strains (see Supplementary Table S4): 12.04% of the 272 ITS2 sites and 1.74% of the mitogenome sites were polymorphic. In addition, the polymorphism rates of 15 conserved protein-coding genes and 2 rRNA genes were varied from 0% to 2.48% (see Supplementary Table S4). Therefore, *L. edodes* sensu lato showed a greater rate of evolution for nuclear genes than for mitochondrial genes. The following question is that which one between nuclear and mitochondrial genes is best for identifying strains. Zietara *et al*.^41^ suggested that both nuclear and mitochondrial genes were necessary to identify the parasite strains of *Gyrodactylus salaries*. In the same sense, we believe that both nuclear and mitochondrial markers are necessary to identify the strains of *L. edodes* sensu lato in China.

To find suitable mitochondrial genes for indentifying the strains of *L. edodes* sensu lato in China, we analyzed the different SNPs among mt-A1, mt-A2 and mt-B and compared four complete mitogenomes with different mitogenome types. These two studies showed some same results as follows: The *cob*, *cox3*, *nad1*, *nad2*, *nad5*, *rps3* and *rrnS* genes had higher diversity between mt-A and mt-B (Fig. 3b) and could distinguish the mitogenome of YAASM363 (mt-B) from the other three mitogenomes (mt-A). In addition, the *cox1* gene may be the biggest difference of 15 conserved protein-coding genes among different mitogenome types. The exon length of *cox1* gene was 1602 bp, but the exon arrangement was distinct among the mitogenomes of mt-A1 (L135 and NC_018365.1), mt-A2 (KY217797.1) and mt-B (YAASM363). Except for the ITS2 sequence, the other nuclear genes should be able to be used to identify the different strains of *L. edodes* sensu lato in China. However, because the same gene was specific in different species, the suitable genes for identifying the two populations of *L. edodes* sensu lato should be determined. In a previous study, we found that the intergenic spacer 1 (IGS1) could be used as a marker for identifying internal mt-A1^42^, and we have sequenced a nuclear genome of YAASM363 (ITS2-B). In the next work, we will determine suitable nuclear markers for strain identification by comparing the nuclear genomes of different ITS2 types.

## Materials and Methods

### Strains, cultivation, DNA isolation and ITS2 sequence analysis of the 20 strains

Twenty strains were assayed in this study (Table 2). These strains were provided by some professional research institutes, were identified by a fruiting test and were maintained at the Institute of Edible Fungi at Shanghai Academy of Agricultural Sciences, Shanghai, People’s Republic of China. The strains were cultured on cellophane PDA (4% potato extract, 20% dextrose and 15% agar) medium in 9 cm Petri dishes at 25 °C in the dark. When growing over the cellophane, the strains were scraped into 5 mL sterile freezing tubes and were freeze-dried by Coolsafe 55-4 (Labogene ScanLaf, Lynge, Denmark). The total DNA of each strain was isolated with the E.Z.N.A Fungal DNA Mini Kit-D3390 (Omega Bio-Tek, Inc, Norcross, United States of America) and was confirmed by 1% (w/v) agarose gel electrophoresis.

All ITS sequences of the twenty strains (Table 2) were used to extract the ITS2 sequences according to the methods described by Song *et al*.^23^. An NJ tree of all ITS2 sequences was reconstructed with MEGA version 7.0. The number of bootstrap replicates was 1000, and a Kimura 2-parameter model was used as the substitution model.

### Illumina library preparation and mitogenome resequencing of the 19 strains

At least 3 μg of the total DNA from each strain was prepared for an Illumina paired-end library. The paired-end library (450 bp) was prepared following the Illumina standard genomic DNA library preparation procedure and was sequenced on an Illumina HiSeq 4000 platform (Illumina, Int, San Diego, United States of America) according to the Illumina sequencing method manual.

Raw Illumina sequencing reads of each strain were generated by CASAVA v 1.8.2 (http://support.illumina.com/sequencing/sequencing_software/casava.ilmn). After removing the adaptors, the high quality reads were obtained using Trimmomatic v 0.36 (http://www.usadellab.org/cms/?page=trimmomatic) and were aligned to the reference genome (L135) using BWA-SW (http://bio-bwa.sourceforge.net/). The BAM file was obtained using Sequence Alignment/Map (SAM) tools v 1.4 (http://samtools.sourceforge.net/). Then, Picard Tools (http://picard.sourceforge.net/) was used to remove the reads caused by PCR-amplification and to obtain the valid BAM files. The depth and coverage of the whole-genome sequencing of each of the other strains (Table 2) were generated by Custom Perl CGI Database Scripts.

### SNP/indel calling, PCA and population structure analysis of the 20 strains

SNPs and indels were detected by the GATK v 3.8.0 “Unified Genotyper” function (http://www.broadinstitute.org/gatk/) based on the valid BAM files and were annotated by ANNOVAR (2017Jul16) (http://www.openbioinformatics.org/annovar/). All SNPs between the reference mitogenome (L135) and the 19 strains were utilized to compute the NJ tree and PCA. The NJ tree was constructed by EMBOSS fneighbor (http://emboss.toulouse.inra.fr/cgi-bin/emboss/fneighbor?_pref_hide_optional=1), and the PCA was performed on the R platform. Population structure analysis was performed using fast structure (https://github.com/rajanil/fastStructure) based on the needed map files, which were generated by PLINK (http://www.plink.com/).

### PacBio library preparation, mitogenome assembly and annotation of L135 and YAASM36

The DNA of L135 and YAASM363 was also used for PacBio library preparation (8–10 kb). A Blue Pippin (Sage Science, Beverly, United States of America) was used for size selection, and the sequencing was performed on the Sequel Sequencer according to manufacturer’s instructions.

The mitogenomes of L135 and YAASM363 were reconstructed from the Illumina and PacBio sequencing data. First, the basic genome framework was assembled from the PacBio data using Celera Assembler v 8.0 (http://sourceforge.net/projects/wgs-assembler/files/wgs-assembler/wgs-8.1/). Second, the basic assembly was verified and the circle mitogenome was completed. Sequencing gaps were filled if there were any. Third, the assembly was corrected and aligned with the Illumina data using Burrows-Wheeler Aligner (BWA) software (http://bio-bwa.sourceforge.net/).

The two mitogenomes were annotated by homology prediction using BLASTn (https://blast.ncbi.nlm.nih.gov/Blast.cgi?PROGRAM=tblastn&PAGE_TYPE=BlastSearch&LINK_LOC=blasthome) and GeneWise (https://www.ebi.ac.uk/Tools/psa/genewise/). The transfer RNA (tRNA) and ribosomal RNA (rRNA) genes were predicted by tRNAscan-SE v 1.4 (http://lowelab.ucsc.edu/tRNAscan-SE/) and by homology prediction (identity >90% and coverage >90%). The secondary structures were predicted with tRNAscan-SE v 1.4 (http://lowelab.ucsc.edu/tRNAscan-SE/). A whole mitochondria genome BLAST search was performed against five databases, including Kyoto Encyclopedia of Genes and Genomes (KEGG), Clusters of Orthologous Groups (COG), Non-Redundant (NR) Protein Database, Swiss-Prot, and Gene Ontology (GO). The circular mitogenome map was drawn using SnapGene v 4.0 (http://www.snapgene.com).

### Synteny analysis and SNP marker screening

Synteny analysis of the four mitogenomes was computed by Mauve (http://darlinglab.org/mauve/user-guide/introduction.html). The population SNP markers and the SNPs from mitochondrial group A were marked in 3 strains with recombinant mitogenomes by Adobe Photoshop CS4 v 11.0.

### Phylogenetic analysis of four complete mitogenomes, conserved protein-coding and rRNA genes

The 15 conserved protein-coding genes and the 2 rRNA genes were extracted from four complete mitogenomes (L135, NC_018365.1, KY217797.1 and YAASM363) and aligned with MEGA version 7.0, respectively. The NJ trees of four complete mitogenomes, 9 conserved protein-coding and 2 rRNA genes (Fig. 4a) were constructed with MEGA version 7.0. The number of bootstrap replicates was 1000, and a Kimura 2-parameter model was used as the substitution model.

### Ethical statement

This article does not contain any studies with human participants or animals performed by any of the authors.

## Supplementary information

Supplementary info

## Data Availability

The L135 strain from mitochondrial group A has been deposited in the Guangdong Microbial Culture Center (GDMCC) under the accession number 5.568, and the mitogenome sequence of L135 has been deposited in GenBank under the accession number MF774812. The YAASM363 strain from mitochondrial group B has been deposited in the GDMCC under the accession number 5.567, and the mitogenome sequence of YAASM363 has been deposited in the GenBank under the accession number MF774813. The final dataset supporting the conclusions of this study is also available, and the raw data are available upon request.
