# Supplementary material for: Mitogenome types of two Lentinula edodes sensu lato populations in China
Source: Sci Rep. 2019 Jul 1;9:9421. doi: 10.1038/s41598-019-45922-5 (PMC6602969; doi:10.1038/s41598-019-45922-5)
Supplement: Supplementary file 1 — Supplementary info [file 41598_2019_45922_MOESM1_ESM.pdf]

Mitogenome types of two *Lentinula edodes* sensu lato populations in China

Xiao-xia Song • Yan Zhao • Chun-yan Song • Ming-jie Chen* • Jian-chun Huang • Da-peng Bao • Qi Tan • Rui-heng Yang

Institute of Edible Fungi, Shanghai Academy of Agricultural Sciences, Shanghai 201403, People's Republic of China

Xiaoxia Song and Yan Zhao contributed equally to this work.

*Corresponding author: Tel: +86 21 62200747; Fax: +86 21 62201337 (M. C.) E-mail: address: mjfungi@126.com (M. C.)

File 1. Features and secondary structures of 26 tRNA genes of L135.

Gene1: *trnP* (32079-32151) Length: 73 bp
Type: Pro Anticodon: TGG at 34-36 (32112-32114) Score: 32.63
* | * | * | * | * | * | * |
Seq:
GTGGTAGAATACCAACGGGtTGGTTACTCCTTTTGGGTGGGAGAGTtAGCGTGTTCGAGTCGCG
CCTATCACG
Str: >>>>>>>...>>>........<<<..>>>>>.......<<<<<.....>>>>>.......<<<<<<<<<<<<.

Gene2: *trnQ* (32673-32746) Length: 74 bp
Type: Gln Anticodon: TTG at 35-37 (32707-32709) Score: 51.31
* | * | * | * | * | * | * |
Seq:
TGAGTTGTAGTTTAATCtGGAAAAACACCATTTTTTGGTAATGGCTAaTATCAGTTCAAATCTG
GTCAACTCAT
Str: >>>>>>>..>>>>.........<<<<.>>>>>.......<<<<<.....>>>>>.......<<<<<<<<<<<<.

Gene3: *trnK* (33512-33582) Length: 71 bp
Type: Lys Anticodon: TTT at 33-35 (33544-33546) Score: 53.30
* | * | * | * | * | * | * |
Seq:
GAAAACAGAACTCGAAGGTTGAGTGTCTCACTTTTAATGAGAAAGTCTTGGTTCAATTCCATG
TGTTTTCA
Str: >>>>>>>..>>>>.......<<<<.>>>>>.......<<<<<....>.>>>.......<<<.<<<<<<<<.

Gene4: *trnS* (34080-34167) Length: 88 bp
Type: Ser Anticodon: TGA at 35-37 (34114-34116) Score: 61.81
* | * | * | * | * | * | * | * | *
Seq:
GGGAATTTGTTAGAGTGGTttAATATAATCGTCTTGAAAACGATTGCTGGAAGAACTTCAGCCG
TGGGTTCGAATCCCACAATTCCCG
Str: >>>>>>>...>>..........<<..>>>>>.......<<<<<.>>>>>>.....<<<<<<.>>>>>.......<<<<<<<<<<<<.

Gene5: *trnE* (34728-34799) Length: 72 bp
Type: Glu Anticodon: TTC at 34-36 (34761-34763) Score: 50.34
* | * | * | * | * | * | * |
Seq:
GATCCTTTAGTATAGTGGTtCGTACAGTTTTCCTTCACAAAACCAGCATCAGTTCGAGTCTGAT
AAGGATCG
Str: >>>>>>>..>>>>........<<<<.>>>>>.......<<<<<....>>>>>.......<<<<<<<<<<<<.

Gene6: *trnS* (34964-35047) Length: 84 bp
Type: Ser Anticodon: GCT at 33-35 (34996-34998) Score: 30.90
* | * | * | * | * | * | * | * |
Seq:
GGAATGGTTTTCATTGGTaAGATACGACAATTGCTAATTGTTTGGTTTAAAACAACCtTGGGTG
TTCGACTCGCCTCCATTCCA
Str: >>>>>>>.>.>>........<<.<.>>>>>.......<<<<<..>>>......<<<...>>>>>.......<<<<<<<<<<<<.

Gene7: *trnW* (60081-60153) Length: 73 bp
Type: Trp Anticodon: CCA at 35-37 (60115-60117) Score: 53.69
* | * | * | * | * | * | * |
Seq:
AAGAGTGTAATATAATTGGTtAGTATATTGATCTCCAAAATCAATCGTAGGTGTTCAAGTCGCC
TCATTCTTG

Str: >>>>>>>..>>>>.........<<<<.>>>>>.......<<<<<....>>>>>.......<<<<<<<<<<<<.

Gene8: *trnD* (61012-61084) Length: 73 bp
Type: AspAnticodon: GTC at 34-36 (61045-61047) Score: 52.91
* | * | * | * | * | * | * |
Seq:
GGGTCCTTAGCTTAATAGGTAGAGCATCTAATTGTCAATTAGGATTgTCCCAGTTCGACTCTGG
AAGGACTCG
Str: >>>>>>>..>>>>........<<<<.>>>>>.......<<<<<......>>>>.......<<<<.<<<<<<<.

Gene9: *trnI* (61172-61242) Length: 71 bp
Type: Ile Anticodon: GAT at 33-35 (61204-61206) Score: 56.15
* | * | * | * | * | * | * |
Seq:
AAGCCAATAATTTAAGGGTAGAATAATTTCTTGATGAGGAATCTGTAGAAGTTCAAATCTTCT
TTGGCTTA
Str: >>>>>>>..>>>>.......<<<<.>>>>>.......<<<<<....>>>>>.......<<<<<<<<<<<<.

Gene10: *trnC* (68233-68308) Length: 76 bp
Type: CysAnticodon: GCA at 36-38 (68268-68270) Score: 25.23
* | * | * | * | * | * | * | *
Seq:
AGAGTTAAGTACAGATGGTtttCTGTAGTAGACCTGCAAAGTCTAACTtTTAGGGTTCAATTCCTT
cTTAACTCTA
Str: >>>>>>>..>>>>..........<<<<.>>>>>.......<<<<<......>>>>.......<<<<..<<<<<<<.

Gene11: *trnR* (68959-69031) Length: 73 bp
Type: ArgAnticodon: TCT at 35-37 (68993-68995) Score: 54.09
* | * | * | * | * | * | * |
Seq:
TTTCTCTTAGTTTAAAAGGTtAGAACTGCAATCTTCTAAATTGCTAATTTTGGTTCAAATCCAAA
AGAGAATA
Str: .>>>>>>..>>>>.........<<<<.>>>>>.......<<<<<....>>>>>.......<<<<<<<<<<<..

Gene12: *trnG* (70450-70522) Length: 73 bp
Type: Gly Anticodon: TCC at 35-37 (70484-70486) Score: 65.46
* | * | * | * | * | * | * |
Seq:
ACAAATATAGTTAAGTGGTctATAACCTCTACCTTCCAAGTAGATAtCATCAGTTCGAATCTGAT
TATTTGTA
Str: >>>>>>>..>>>>.........<<<<.>>>>>.......<<<<<....>>>>>.......<<<<<<<<<<<<.

Gene13: *trnG* (70529-70601) Length: 73 bp
Type: Gly Anticodon: TCC at 35-37 (70563-70565) Score: 65.21
* | * | * | * | * | * | * |
Seq:
ACGAATATGGTTAAGTGGTctATAACCTCTACCTTCCAAGTAGATAtCATCAGTTCGAATCTGAT
TATTCGTA
Str: >>>>>>>..>>>>.........<<<<.>>>>>.......<<<<<....>>>>>.......<<<<<<<<<<<<.

Gene14: *trnH* (71314-71384) Length: 71 bp
Type: His Anticodon: GTG at 33-35 (71346-71348) Score: 54.60
* | * | * | * | * | * | * |
Seq:
GTAGAATTAGTTTAATGGTAAAATGACGTCTTGTGGCGACGATGtTCAGAGTTCGAGTCTCTGA
TTCTACC

Str: >>>>>>>..>>>>.......<<<<..>>>>.......<<<<.....>>>>>.......<<<<<<<<<<<<.

Gene15: *trnV* (82746-82817)   Length: 72 bp
Type: Val Anticodon: TAC at 34-36 (82779-82781) Score: 60.30
* | * | * | * | * | * | * |
Seq:
AGGGAATTAGCTGAGTGGTtTTAGCATCAATTTTACACATTGGAGACAGAGTTTCGATTACTCT
ATTCCCTA
Str: >>>>>>>..>>>>........<<<<.>>>>>.......<<<<<....>>>>>.......<<<<<<<<<<<<.

Gene16: *trnL* (83281-83369)   Length: 89 bp
Type: Leu Anticodon: TAG at 35-37 (83315-83317) Score: 48.21
* | * | * | * | * | * | * | * | *
Seq:
ACGAGTATGCCGAAATTGGTaGACGGGTGAGTTTTAGGTACTCATGATTTTTTTTTTTAATTGT
AAGAGTTCAAGTCTCTTTATTCGTA
Str: >>>>>>>..>>>...........<<<.>>>>>.......<<<<<..>.>>........<<.<..>>>>>.......<<<<<<<<<<<<.

Gene17: *trnR* (83450-83522)   Length: 73 bp
Type: Arg Anticodon: TCG at 34-36 (83483-83485) Score: 62.59
* | * | * | * | * | * | * |
Seq:
GCATCTTTAGCTGAATTGGTACAGCAATTGCCTTCGAAGCAAGAGTtTATTGGTTCGAGTCCGA
TAAGATGTT
Str: >>>>>>>..>>>>........<<<<..>>>>.......<<<<.....>>>>>.......<<<<<<<<<<<<.

Gene18: *trnA* (84296-84368)   Length: 73 bp
Type: Ala Anticodon: TGC at 34-36 (84329-84331) Score: 55.49
* | * | * | * | * | * | * |
Seq:
GGGGAAATACGATAATTGGTAATCAGTTATATTTGCAATATAAAAAaTGATAGTTCGAGTCTA
TCTTTCTCCA
Str: >>>>>>>...>>>........<<<..>>>>>.......<<<<<.....>>>>>.......<<<<<<<<<<<<.

Gene19: *trnF* (84800-84871)   Length: 72 bp
Type: Phe Anticodon: GAA at 34-36 (84833-84835) Score: 65.82
* | * | * | * | * | * | * |
Seq:
GCCCCGGTAGCTTAGTGGTaAAAGCGTTATATTGAAGCTATAGAAATGGGAGTTCAATTCTCT
CCCGGGGCA
Str: >>>>>>>..>>>>........<<<<.>>>>>.......<<<<<....>>>>>.......<<<<<<<<<<<<.

Gene20: *trnT* (85384-85455)   Length: 72 bp
Type: Thr Anticodon: TGT at 34-36 (85417-85419) Score: 56.53
* | * | * | * | * | * | * |
Seq:
GTCGAAATAGTTTAATTGGTAAAACAAATTCCTTGTAAGATTTAAATATTGGTTCAATTCCATT
TTTCGGCT
Str: >>>>>>>..>>>>........<<<<.>>.>>.......<<.<<....>.>>>.......<<<.<<<<<<<<<.

Gene21: *trnM* (85668-85740)   Length: 73 bp
Type: Met Anticodon: CAT at 35-37 (85702-85704) Score: 58.02
* | * | * | * | * | * | * |
Seq:
GCTGAAATAGTTTAAATGGTcAAAACATACCATTCATGACGGTAAAAAGAAGGTTCAAATCCT
TCTTTCGGCT

Str: >>>>>>>..>>>>.........<<<<.>>>>.........<<<<....>>>>>.......<<<<<<<<<<<<.

Gene22: *trnM* (107066-107137)   Length: 72 bp
Type: MetAnticodon: CAT at 33-35 (107098-107100)   Score: 56.83
* | * | * | * | * | * | * |
Seq:
AAGACTTTAGCATAACGGTAATGCAATTCGCTCATAATGGATAAGaTAAAGGTTCGATTCCTTT
AGGTCTTA
Str: >>>>>>>..>>>>.......<<<<.>>>>>.......<<<<<.....>>>>>.......<<<<<<<<<<<<.

Gene23: *trnY* (107743-107826)   Length: 84 bp
Type: Tyr Anticodon: GTA at 35-37 (107777-107779)   Score: 38.61
* | * | * | * | * | * | * | * |
Seq:
GGGTAGGTgATCCGATTGGTaATGGTGGTTCTCTGTAAAAGAATTGGTTAATAACCGtAAGAGG
TTCGATTCCTCTACTACTCA
Str: >>>>>>.....>>>........<<<..>>>>>.......<<<<<.>>>>...<<<<...>>>>>.......<<<<<.<<<<<<.

Gene24: *trnN* (108633-108705)   Length: 73 bp
Type: AsnAnticodon: GTT at 34-36 (108666-108668)   Score: 57.48
* | * | * | * | * | * | * |
Seq:
GGTTCTATGGCTGAGTGGTtTAAGCGAAGTACTGTTAATACTTTTTaCAGAGGTTCAAATCCTCT
TGGGGCCT
Str: >>>>>>>..>>>..........<<<.>>>>>.......<<<<<.....>>>>>.......<<<<<<<<<<<<.

Gene25: *trnL* (109774-109856)   Length: 83 bp
Type: LeuAnticodon: TAA at 34-36 (109807-109809)   Score: 52.42
* | * | * | * | * | * | * | * |
Seq:
GCGAACATGTTGAAATGGTaAACAATCTCCTCTTAAGCAGGAGTGGAAGTGATTCCtTAAGAGT
TCAAGTCTCTTTGTTCGTA
Str: >>>>>>>..>>>..........<<<.>>>>>.......<<<<<..>>>.><.<<<...>>>>>.......<<<<<<<<<<<<.

Gene26: *trnM* (110261-110333)   Length: 73 bp
Type: MetAnticodon: CAT at 35-37 (110295-110297)   Score: 59.74
* | * | * | * | * | * | * |
Seq:
GGCGAGATGGTGTAATTGGAtAGCACTAAAGTCTCATTAGCTTTGGGTTTAGGTTCAAATCCTA
ATCTCGCCA
Str: >>>>>>>..>>>>.........<<<<.>>>>>.......<<<<<....>>>>>.......<<<<<<<<<<<<.

Table S1. Resequencing the mitogenomes of 19 strains.

Strain	Reads(×10^6)	Q20（%）	Q30(%)	Mapped Reads(×10^5)	Mapped rate (%)	Genome coverage (%)	Average depth
Cr01	8.28	97.96	93.82	4.60	5.56	100.00	563.48
EFISAAS0376	7.00	97.95	93.83	5.17	7.39	89.08	623.76
EFISAAS5052	33.40	97.49	92.23	16.58	4.96	97.95	2014.46
EFISAAS5053	7.79	97.84	93.54	4.45	5.71	97.74	544.20
EFISAAS5054	6.88	97.85	93.58	6.12	8.90	98.23	747.57
EFISAAS5145	29.84	97.58	92.42	13.24	4.44	90.39	1579.84
EFISAAS5143	6.69	97.86	93.60	6.01	8.98	100.00	737.90
EFISAAS5146	6.85	97.80	93.44	7.72	11.27	99.86	947.85
Guangxiang No.9	31.96	97.43	92.02	13.90	4.35	99.99	1687.82
L808	28.19	97.39	91.91	6.33	2.25	100	770.96
YAASM296	9.00	97.93	93.77	5.20	5.78	87.49	627.73
YAASM298	8.41	97.87	93.64	4.72	5.62	93.82	569.12
YAASM300	9.51	97.87	93.62	4.63	4.88	89.05	558.08
YAASM301	28.98	97.28	91.71	9.83	3.39	92.54	1167.22
YAASM359	35.53	97.58	92.40	8.39	2.36	90.39	999.29
YAASM363	33.62	97.61	92.47	14.09	4.19	90.43	1687.13
YAASM1515	39.15	97.55	92.34	17.46	4.46	94.00	2084.29
YAASM2321	41.26	97.68	92.72	30.90	7.49	99.73	3670.04
YAASM2323	9.95	97.95	93.81	5.35	5.38	96.10	649.97

Table S2. SNP diversity in the CDS regions of the mitogenomes of 19 resequenced strains aligned with L135.

Population type	Strain	CDS of 30 hypothetical protein-coding genes				CDS of 15 conserved protein-coding genes			
		N	S	G	L	N	S	G	L
A1	Cr01	14	6	3	1	1	2	0	0
	EFISAAS5143	11	4	0	1	1	2	0	0
	EFISAAS5146	7	5	2	1	4	2	0	0
	L808	12	2	0	1	1	0	0	0
	Average	*11b*	*4b*	*1a*	*1a*	*2b*	*2b*	*0a*	*0a*
A2	EFISAAS5052	10	5	1	1	2	4	0	0
	EFISAAS5053	14	6	1	1	1	4	0	0
	EFISAAS5054	14	6	1	1	1	4	0	0
	Guangxiang No.9	14	5	2	1	1	3	0	0
	Average	*13b*	*5b*	*1a*	*1a*	*1b*	*4b*	*0a*	*0a*
B	EFISAAS0376	76	25	1	1	15	32	0	0
	EFISAAS5145	74	19	0	1	16	32	0	0
	YAASM296	70	25	0	1	16	33	0	0
	YAASM298	71	21	0	2	16	23	1	0
	YAASM300	71	23	0	1	15	33	0	0
	YAASM301	75	25	1	1	13	23	1	0
	YAASM359	68	25	0	2	16	32	0	0
	YAASM363	62	22	0	2	17	33	0	0
	YAASM1515	78	22	0	2	17	33	0	0
	YAASM2321	71	23	0	2	19	32	0	0
	YAASM2323	74	28	1	2	15	31	0	0
	Average	*72a*	*23a*	*0a*	*2a*	*16a*	*31a*	*0a*	*0a*

Notes: The different lowercase letters indicate significant differences at the 0.05 level.
N: Nonsynonymous mutation; S: Synonymous mutation; G: Stopgain; L: Stoploss.

Table S3. Mitogenome annotations of L135, NC_018365.1, KY217797.1 and YAASM363.

Gene	The number of bases			
	L135	NC_018365.1	KY217797.1	YAASM363
cob	8398	8389	8463	8593
cob-exon1	156	156	*156*	156
cob-exon2	27	27	*27*	27
cob-exon3	228	228	*228*	228
cob-exon4	759	759	759	759
orf240	723	*723*	*723*	723
cox2	759	759	*759*	759
orf715	2148	*2148*	*2148*	2127(orf708)
nad6	627	627	627	627
atp6	768	768	768	768
cox3	2280	2280	*2280*	2256
cox3-exon1	465	465	*465*	471*
cox3-exon2	366	366	*366*	366
orf223	672	672	*672*	672
orf575	1728	*1728*	*1728*	1734(orf577)
nad4L	267	267	267	267
nad5	3457	3457	3457	3470
nad5-exon1	936	936	*936*	936
nad5-exon1	1113	1113	*1113*	1113
orf456	1371	*1356(orf451)*	*1371*	1371
trnP	73	73	73	73
trnQ	74	76	74	74
trnK	71	71	71	71
trnS	88	88	88	88
trnE	72	74	72	72
trnS	84	84	84	84
cox1	11088	11088	8391	12029
cox1-exon1	234	234	234	234
cox1-exon2	150	150	684^	480^
cox1-exon3	330	330		204
cox1-exon4	204	204		36^
cox1-exon5	132	132	132	96^
cox1-exon6	51	51	51	51
cox1-exon7	198	198	198	198
cox1-exon8	303	303	303	303
orf202	609	609	/	609
orf440	1323	1323	/	/
orf288	867	867	/	873(orf290)
orf214	645	645	/	645
orf271	/	/	/	816
orf367	1104	1104	/	1098(orf365)
orf255	768	*768*	*786*	768
orf190	573	573	/	882(orf293)
orf212	639	*639*	*639*	720(orf239)
rrnL	9884	9871	*9898*	9931
rrnL-exon1	1321	*1321*	*1321*	1321
rrnL-exon2	1183	*1183*	*1183*	1183
rrnL-exon3	308	*308*	*308*	308
rrnL-exon4	230	*230*	*230*	230
rrnL-exon5	234	*234*	*234*	234
rrnL-exon6	627	*627*	*627*	627

orf324	975	975	*975*	981(orf326)
orf196	591	591	372(orf123)	408(orf135)
orf365	1098	1098	*1098*	1104(orf367)
trnW	73	73	73	73
trnD	73	73	73	73
trnI	71	73	71	71
Misc_feature	1895	1898	*1895*	1895
orf267	/	/	/	804
orf261	786	*789(orf262)*	*786*	786
orf379	/	*/*	/	1140
orf113	342	*342*	*342*	/
atp8	159	159	159	159
trnC	76	76	76	76
trnR	73	75	73	73
trnG	73	73	73	73
trnG	73	73	73	73
orf138	417	*417*	*327(orf108)*	330(orf109)
trnH	71	73	71	71
rps3	4407	4407	*4407*	4407
rrnS	3514	3514	*3505*	2166^
rrnS-1	1330	1330	*1330*	/
rrnS-2	833	833	*833*	/
orf401	1206	1206	*1074(orf357)*	/
trnV	72	72	72	72
trnL	89	89	89	86
trnR	73	73	73	73
trnA	73	73	73	73
trnF	72	72	72	72
trnT	72	74	72	72
trnM	73	73	73	73
nad2	1935	1935	*1935*	1935
nad3	384	384	384	384
orf206	621	*621*	*621*	606 (orf201)
orf99	300	*300*	*300*	/
atp9	222	222	222	222
nad4	1464	1464	1464	1464
Misc_feature	3079	3079	*3073*	/
orf131	396	*396*	*396*	/
orf272	819	*819*	*819*	/
orf181	546	*546*	420(orf139)	/
orf154	465	*465*	*465*	/
orf101	306	*306*	306	/
orf130	393	*394*	*393*	/
orf283	852	*852*	*435(orf144)*	/
Misc_feature	2281	2287	*2287*	2264
trnM	72	72	72	72
trnY	84	85	84	84
trnN	73	73	73	73
trnL	83	84	83	83
trnM	73	73	73	73
nad1	4887	4850	4880	4792
nad1-exon1	153	153	153	153
nad1-exon2	144	144	144	144
nad1-exon3	360	360	*360*	360
nad1-exon4	360	360	360	360
orf184	555	555	*555*	555

Notes: A number in italics indicates genes that are not annotated or incorrect annotated in NC_018365.1 or KY217797.1 but were inferred by aligning the sequences with those of L135. / indicates no homologous genes. * indicates insertion. ^ indicates alternative splicing. _ indicates intronic hypothetical protein-coding genes.

Table S4. The polymorphism rates of ITS2, the whole mitogenome and some mitochondrial genes between L135 and the other 19 strains

Sequence	Length of ITS2, mitogenome or mitochondrial genes of L135 (bp)	No. of polymorphism site	Polymorphism rate (%)
ITS2	274	33	12.04
mitogenome	119134	2071	1.74
cob	8398	208	2.48
cox2	759	3	0.40
nad6	627	1	0.16
atp6	768	0	0
cox3	2280	14	0.61
nad4L	267	0	0
nad5	3457	29	0.84
cox1	11088	53	0.48
atp8	159	0	0
nad2	1935	23	1.19
nad3	384	2	0.52
atp9	222	0	0
nad4	1464	1	0.07
nad1	4887	67	1.37
rps3	4407	20	0.45
rrnL	9884	111	1.12
rrnS	3514	29	0.83

Site	13	15	20	Gene	Site	13	15	20	Gene	Site	13	15	20	Gene
3844		•		*cob* -intron	33757		•		/	39503		•		*cox1* -intron
6597		•	•		33800		•	•		39534		•		*cox1* -exon
9306	•	•		/	33910		•			39935		•		orf288
9616		•	•		33940		•			39978		•		
10919			•		34032		•			40161		•		
13066			•	orf715	34033		•			40212		•		
13815			•		34062		•			40305		•		
14246			•	/	34236		•			40392		•		
14337			•		34287		•			40447		•		
14479			•		34297		•			40512		•		
14558			•		34311		•			40527		•		
14572			•		34313		•			40596		•		
14703			•		34351		•			41224		•		orf214
14759			•		34373		•			41339		•		
14781			•		34402		•			42039		•		*cox1* -intron
14827			•		34452		•			42245		•		
14939			•		34466		•			42304	•	•	•	
15000	•		•		34473		•			46052		•		
15084			•		34476		•			46074	•	•		
15115			•		34504		•			49278	•	•	•	/
15186			•		34560		•			49527		•		
15439			•		34577		•			49541		•		
15550			•		34579		•			49995	•	•	•	*rrnL*
15554			•		34601		•			49997	•	•	•	
15908			•		34694		•			52656		•	•	
15969			•		34825		•			53131	•	•	•	orf324
17288		•			34853		•			54239		•	•	orf196
17515	•	•			34858		•			54590			•	
17845		•			34870		•			54867			•	*rrnL*
18519			•		34897		•			55745	•	•		
21906		•		*cox3* -exon	35099		•			55859	•	•		orf365
22072		•		*cox3* -intron	35101		•			55881	•	•		
22846		•	•		35212		•			56008	•	•		
24533		•		orf575	35241		•			56914		•	•	
24537		•			35310		•			58332		•	•	*rrnL*
25023		•			35387		•			59744			•	/
25658		•		/	35634		•			59789			•	
26693			•	*nad5* -exon	35679		•			59829	•	•	•	
26922			•	*nad5* -intron	35700		•			59845			•	
27225			•		35701		•			59856	•		•	
27237			•		35711		•			60061			•	
27258		•			35712		•			60284			•	
27786	•				35730		•			60459			•	
27878			•		35732		•			60610			•	
27996			•		35734		•			62169			•	
31563		•		orf456	35739		•			62228			•	
31712		•			35838		•			62229			•	
31787		•		/	35910		•		*cox1* -exon	62230			•	
32266		•	•		36124		•		*cox1* -intron	62257			•	
32267		•	•		36144		•			62271			•	
32291		•			36146		•			62378			•	
32384		•			36358		•			62411			•	
32386		•			36477		•		orf202	62460			•	
32476	•	•			36593		•			62530			•	
33000		•			36602		•			62661			•	
33012		•			36653		•			62821			•	
33013		•			36908		•			62870			•	
33014		•			37159		•		*cox1* -intron	62903			•	
33027		•			37166		•			63026			•	orf261
33041		•			37171		•			63035			•	
33136		•			37176		•			63158			•	
33150		•			37178		•			63183			•	
33177		•			37184	•		•		63275			•	
33232		•			37207		•			63629			•	
33241		•			37218	•		•		63634			•	
33243		•			37236		•	•		63651			•	
33295		•			37244		•			63659			•	
33318		•			37246		•			63920			•	/
33442		•			37247		•			64077			•	
33452		•			37265		•			64133			•	
33662		•			37764		•		*cox1* -exon	64234			•	
33697		•			37777		•		*cox1* -intron	64245	•	•	•	
33719		•			39137		•			64246	•	•	•	
33754		•			39502		•			64614			•	

Site	13	15	20	Gene	Site	13	15	20	Gene	Site	13	15	20	Gene
64705			•	/	74169		•	•	*rps3*	84409	•			/
64885			•		75934		•			84416	•			
64991			•		77553		•		/	84431	•			
65037			•		77582		•			84447	•			
65387			•		77586		•			84461	•			
65477		•	•		77587		•			84522	•			
65551	•		•		77606		•			84537	•			
65586	•		•		77612		•			84594	•			
65598	•		•		77613		•			84601	•			
65622		•	•		77631		•			84623	•			
65688	•		•		78756		•	•		84656	•			
65699		•	•		80024		•		*rrnS*	84688	•			
65787			•		80213		•			84709	•	•		
65788	•		•		81403		•		orf401	84739	•			
65814	•		•		81562		•		*rrnS*	84749	•			
65875	•		•		81566		•			84782	•			
65879		•	•		81811		•			84789	•			
66029	•		•		81826		•			84926		•		
66053	•		•		81903		•			85008	•			
66059	•		•		82401	•	•		/	85069	•			
66078	•		•		82418	•				85074	•			
66151		•			82424	•				85079	•			
66199		•			82426	•				85083		•		
66364		•			82468	•				85087	•			
66403	•		•		82480	•				85094	•			
66566	•		•		82484	•				85095		•		
66568	•		•		82546	•				85096	•			
66605	•		•		82548	•				85099		•		
66617	•		•		82552	•				85109	•	•		
66707	•		•		82561	•				85116	•	•		
66737	•		•		82589	•				85117	•			
66762		•			82594	•				85152	•			
66769		•			82602	•				85161	•			
66784		•			82605	•				85170	•			
66794	•		•		82606	•				85181	•			
66797	•		•		82609	•				85187	•			
66801	•		•		82634	•				85206	•			
66805	•		•		82643	•				85216	•			
66806	•		•		82657	•				85217	•			
66827	•		•		82703	•				85220	•			
66829	•		•		82727	•				85225	•			
66964	•		•		82853	•				85241	•			
66966	•		•		82909	•				85250	•			
67012	•		•		82981	•				85255	•			
67037	•		•		82989	•				85299	•			
67042	•		•		83012	•				85925	•	•		
67043	•		•		83021	•				86133	•	•		
67044	•		•		83071	•				86137	•	•		
67045	•		•		83075	•				86139	•	•		
67050	•		•		83083	•				86147	•	•		
67051	•		•		83102	•				86152	•	•		
67054	•		•		83293	•				86167		•		
67068		•			83387	•				86168	•	•		
67075		•			83548	•				86169		•		
67081	•		•		83599	•				86173	•	•		
67326			•		83753	•				86257		•		
67487			•		83754	•				86268	•			
67985		•	•		83776	•				86326	•			
68475		•			83777	•				86448	•	•		
68799		•			83812	•				86459	•	•		
68800		•			83829	•		•		86476	•	•		
69192	•		•		83830	•				86490	•	•		
69544			•		83839	•		•		86492	•	•		
69547	•	•			83860	•				86493	•	•		
69551			•		84040	•				86622	•	•		
69657		•			84137	•				86650	•	•		
70053		•	•		84195	•				86832	•	•		
71138	•	•			84196	•				86851	•	•		
71857		•	•		84205	•				86899	•	•		
72530		•			84208	•				86923	•	•		
72532		•			84217	•				86929	•	•		
72641		•	•		84230	•				86941	•	•		
73741	•		•	*rps3*	84281	•				87015	•	•		
74165		•	•		84394	•				87019	•	•		

Site	13	15	20	Gene	Site	13	15	20	Gene	Site	13	15	20	Gene
87033	•	•		/	96593			•	/	108137			•	/
87093	•	•			96607			•		108379		•	•	
87141	•	•			96626			•		108764			•	
87143	•	•			96638			•		108946			•	
87154	•	•			96759			•		109072			•	
87170	•	•			96906			•		109075			•	
87197	•	•			96912			•		109245		•	•	
87235	•	•			97041			•		109257		•	•	
87256	•	•			97102			•		109266			•	
87259	•	•			97124			•		109370			•	
87275	•	•			97130			•		109573		•	•	
87277	•	•			98041			•	orf272	109727			•	
87278	•	•			98197			•		110044			•	
87290	•	•			98732			•		110091			•	
87296	•	•			98784			•		110157			•	
87313	•	•			99000			•	orf181	110176			•	
87314	•	•			99409			•		110187			•	
87340	•	•			99565			•	/	110209			•	
87354	•	•			99855			•		110246			•	
87370	•	•			100288			•	orf154	110366			•	
87375	•	•			100455			•	/	110598			•	
87380	•	•			100456			•		110657			•	
87624	•	•		*nad2*	100916			•		110671			•	
87730	•	•			101881			•		110977			•	
87793	•	•			102271			•		111079			•	
87928	•	•			102331			•		111173			•	
88169	•	•			102341			•		111178			•	
88396	•	•			102691			•	orf130	111181			•	
88513	•	•			102747			•		111198			•	
88549	•	•			102867			•		111218			•	
89068	•	•			103664			•	orf283	111219		•	•	
89155	•	•			104266			•		111230			•	
89180	•	•			104322			•	/	111249			•	
89192	•	•			104381			•		111259			•	
89538	•	•		*nad3*	104385			•		111271			•	
89547	•	•			104414			•		111272			•	
89737	•	•		/	104510			•		111280			•	
89741	•	•			104553			•		111290			•	
89746	•	•			104622			•		111420			•	
89758	•	•			104639			•		111440			•	
89783	•	•			104803			•		111444			•	
89795	•	•			104967			•		111524			•	
89800	•	•			105129			•		111822			•	
89808	•	•			105271			•		111892			•	
89841	•	•			105297			•		112098			•	
89858	•	•			105298			•		112107			•	
89908	•	•			105303			•		112130			•	
89984	•	•			105304			•		112150			•	
90017	•	•			105307			•		112151			•	
90065	•	•			105339			•		112155			•	
90074	•	•			105846			•		112159			•	
90156		•			105896			•		112164			•	
90222	•	•			106052			•		112232			•	
90223	•				106253			•		112295			•	
90984	•				106305			•		112305			•	
91192	•			orf99	106347			•		112340	•		•	
91875	•	•	•	/	106503			•		112343	•		•	
93224			•		106819			•		112352			•	
93860			•		106830			•		112356			•	
93908			•		106831			•		112361	•		•	
94004	•	•	•		106846			•		112362			•	
94338			•		107035			•		113435	•			*nad1* -intron
94434			•		107186			•		114407		•		
94629	•	•	•		107187			•		115542		•	•	
94664			•		107191			•		116494			•	
94667			•		107204		•	•		117544		•	•	/
94940			•		107205		•	•		117570		•	•	
94958			•		107245			•		117595		•	•	
94962			•		107251			•						
95016			•		107485			•						
95019			•		107904			•						
95029			•		107934			•						
96561			•	*nad4*	107944			•						
96584			•	/	108005			•						

Fig. S1 SNPs from mt-A in 3 strains with recombinant mitogenome.

13: YAASM298, 15: YAASM301, 20: YAASM2323. / indicates intergenic regions.- indicates a base deletion.
